# Fructose Intake Is Associated with Brain Metabolic Reprogramming and Exacerbation of Alzheimer-like Alterations in APP/PS1 Mice

**DOI:** 10.3390/ijms27094113

**Published:** 2026-05-04

**Authors:** Paulina Ormazabal, Patricio Órdenes-Constenla, Camila Gherardelli, Marianela Bastías-Pérez, José Brito-Valenzuela, Marcelo Flores-Opazo, Nibaldo C. Inestrosa, Pedro Cisternas

**Affiliations:** 1 Facultad de Ciencias para el Cuidado de la Salud, Universidad San Sebastián, Lota 2465, Providencia, Santiago 8420524, Chile; paulina.ormazabal@uss.cl; 2 Facultad de Ciencias, Universidad San Sebastián, Concepción 4030000, Chile; patricio.ordenes@uss.cl; 3 Mitchell Center for Alzheimer’s Disease and Related Brain Disorders, Department of Neurology, University of Texas McGovern Medical School, Houston, TX 77030, USA; cgherardelli@gmail.com; 4 Centro de Investigación Biomédica en Red (CIBER) de Fisiopatología de la Obesidad y Nutrición (CIBEROBN), Instituto de Salud Carlos III (ISCIII), E-28029 Madrid, Spain; mbastias@udla.cl; 5 Núcleo de Investigación en Nutrición y Ciencias Alimentarias (NINCAL), Facultad de Salud y Ciencias Sociales, Universidad de Las Américas, Santiago 1530000, Chile; 6 Departamento de Obstetricia y Puericultura, Facultad de Medicina, Universidad de Concepción, Concepción 4030000, Chile; jobrito@udec.cl; 7 Musculoskeletal Rehabilitation Laboratory, Institute Exercise and Rehabilitation Sciences (ICER), Universidad Andrés Bello, Campus Casona, Las Condes, 7550000, Chile; marcelo.flores@unab.cl; 8 Centro de Excelencia en Biomedicina de Magallanes (CEBIMA), Escuela de Medicina, Universidad de Magallanes, Punta Arenas 6200000, Chile; 9 Facultad de Ciencias Biológicas, Pontificia Universidad Católica de Chile, Av. Bernardo O’Higgins 340, Santiago 7820436, Chile

**Keywords:** fructose, Alzheimer’s disease, glucose metabolism, metabolic syndrome, cognitive decline

## Abstract

Emerging evidence implicates metabolic dysfunction as a key contributor to Alzheimer’s disease (AD) pathogenesis. Fructose, a major component of modern diets, promotes systemic metabolic alterations; however, its direct impact on AD-related brain dysfunction remains poorly defined. Here, we investigated the effects of short-term fructose consumption on systemic metabolism, brain glucose handling, and cognitive performance in APP/PS1 transgenic mice. Six-month-old asymptomatic male mice received 15% fructose in drinking water for eight weeks, while controls received plain water. Fructose-fed APP/PS1 mice developed metabolic alterations consistent with early metabolic syndrome, including increased fasting glucose and dyslipidemia. These changes were accompanied by reduced cerebral glucose utilization, increased Aβ_42_ accumulation, and impaired cognitive performance. In parallel, fructose intake enhanced neuroinflammatory markers, suggesting a coordinated disruption of metabolic and inflammatory pathways in the brain. Collectively, these findings support the idea that fructose consumption may exacerbate Alzheimer-like alterations linking systemic metabolic dysfunction to impaired brain glucose metabolism and neuroinflammation. This study provides mechanistic evidence supporting a role for dietary fructose as a modifiable risk factor in AD vulnerability.

## 1. Introduction

The global burden of Alzheimer’s disease (AD) continues to increase, driven not only by aging but also by lifestyle-related factors that disrupt metabolic homeostasis [1,2]. Growing evidence indicates that metabolic disorders, including obesity, insulin resistance, and type 2 diabetes, significantly elevate the risk of developing AD and may accelerate its progression [3,4]. These associations have led to the concept of AD as a disorder closely linked to impaired glucose metabolism, oxidative stress, and neuroinflammation [5,6,7].

Among the key dietary contributors to modern metabolic imbalance is fructose, a monosaccharide widely used as a sweetener in processed foods and beverages [8]. High fructose intake promotes hepatic lipogenesis, insulin resistance, and systemic inflammation, features collectively defining the metabolic syndrome (MetS) [9,10,11]. While its peripheral effects are well documented, less is known about how fructose impacts brain metabolism and cognitive integrity, particularly under conditions of genetic vulnerability such as those present in AD [12,13,14,15].

We previously demonstrated that fructose consumption disrupts hippocampal synaptic plasticity and impairs spatial memory in healthy mice, in association with oxidative stress and lipid peroxidation [13]. However, whether fructose-induced metabolic stress exacerbates neuropathological features in genetically predisposed models of AD remains unclear [12,15]. 

Although excessive fructose consumption has been linked to systemic metabolic dysfunction, its direct impact on brain energy metabolism and its potential contribution to AD-related pathology remain insufficiently understood. Here, we investigated whether chronic fructose intake alters brain glucose metabolism and exacerbates AD-like features in APP/PS1 mice.

The APP/PS1 transgenic mouse represents a well-established model of amyloid β (Aβ) accumulation and early cognitive decline [16,17,18]. In these animals, amyloid deposition begins around 7–8 months of age, providing a window to investigate environmental modifiers during the pre-symptomatic stage [19].

Here, we examined whether fructose exposure in asymptomatic APP/PS1 mice would precipitate systemic metabolic alterations and exacerbate central neuropathology. To test this, six-month-old APP/PS1 male mice received 15% fructose in drinking water for eight weeks, a concentration comparable to human dietary consumption levels associated with metabolic dysfunction [13,20]. The 15% (*w*/*v*) fructose concentration was selected based on previous experimental models demonstrating that this range (10–20%) reliably induces insulin resistance, dyslipidemia, and metabolic syndrome-like features in rodents without overt toxicity. Importantly, this concentration approximates the relative fructose load observed in Western-style diets characterized by high intake of sugar-sweetened beverages and processed foods, which may contribute up to 15–25% of total caloric intake in susceptible populations [21]. Furthermore, we have previously shown that 15% fructose in drinking water is sufficient to disrupt hippocampal synaptic plasticity and cognitive performance in wild-type (WT) mice, supporting its translational and mechanistic relevance in metabolic-brain interaction studies [13,21].

Control animals received water only. At eight months of age, when amyloid deposition becomes detectable, we evaluated (i) peripheral metabolic parameters including glucose, triglycerides, cholesterol, insulin, and insulin resistance; (ii) behavioral performance using novel object recognition (NOR) and localization (NOL) tests; (iii) brain glucose accumulation via radiolabeled glucose tracing; (iv) amyloid pathology through Aβ_1-42_ accumulation; (v) mRNA levels of inflammatory cytokines and (vi) metabolic markers related to glycolysis and the pentose phosphate pathway (PPP).

Our findings reveal that exposure to fructose markedly alters systemic metabolism and precipitates cognitive and molecular features reminiscent of AD pathology. Specifically, fructose-fed APP/PS1 mice displayed increased Aβ_1-42_ deposition, higher Tumor necrosis factor (TNF-α) and Interleukin 6 (IL-6) mRNA levels, reduced glucose accumulation in the brain, and behavioral deficits in cognitive performance. Notably, these central alterations occurred in parallel with elevated insulin resistance (HOMA index) and dyslipidemia, despite the absence of hepatic injury. These findings led us to test whether dietary fructose could exacerbate AD-like alterations by coupling peripheral metabolic stress with central neuroinflammatory and metabolic dysfunction.

In summary, this study shows that short-term fructose exposure in APP/PS1 mice promotes a metabolic syndrome-like phenotype and aggravates key pathological features of AD, including cognitive impairment, amyloid accumulation, and neuroinflammation. These findings strengthen the concept that metabolic health is inseparable from brain health and reinforce the urgency of preventive dietary interventions in mitigating the growing epidemic of AD.

## 2. Results

### 2.1. Fructose Supplementation Induces Metabolic Alterations Consistent with a Metabolic Syndrome-like Phenotype

**Figure 1:**
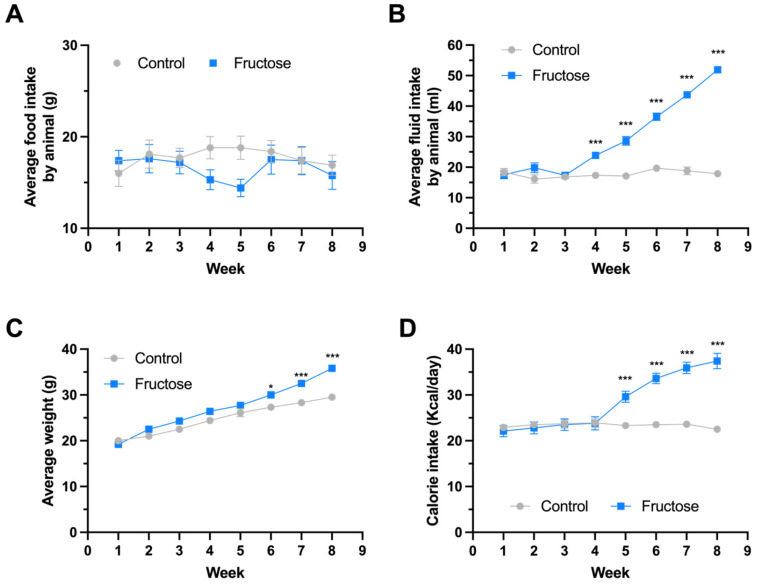
Fructose intake alters energy balance and promotes weight gain in APP/PS1 mice. Effects of fructose consumption on food intake, fluid intake, body weight, and caloric intake in APP/PS1 mice. (**A**) Average weekly solid food intake per animal. (**B**) Average weekly fluid intake per animal. (**C**) Body weight progression during the 8-week intervention. (**D**) Total caloric intake calculated from solid food and liquid consumption. Mice received either standard drinking water (Control) or 15% fructose solution (Fructose) for 8 weeks. Data are presented as mean ± SEM. Statistical significance was determined by two-way ANOVA with repeated measures followed by post hoc analysis. * *p* ≤ 0.05, *** *p* < 0.001 vs. Control.

To assess the systemic impact of fructose exposure in APP/PS1 mice, we monitored food intake, fluid intake, average weight and calorie intake over an eight-week period. Average food intake remained stable between control and fructose-fed mice throughout the experiment, indicating that caloric differences were not attributable to reduced feeding behavior (Figure 1A). In contrast, animals receiving fructose solution exhibited a progressive and significant increase in fluid consumption beginning at week 4 (Figure 1B), consistent with the high palatability of the sweetened solution. This rise in liquid intake was paralleled by a slight increase in body weight (*p* < 0.05 at week 4; *p* < 0.001 thereafter; Figure 1C). Consequently, daily caloric intake was significantly elevated in the fructose group compared with controls from week 5 through week 8 (*p* < 0.001; Figure 1D). These findings indicate that short-term fructose supplementation promotes positive energy balance without altering solid food consumption.

Consistent with these physiological changes, biochemical analysis revealed a metabolic profile reminiscent of early metabolic syndrome (Table 1). Fructose-fed mice showed significantly elevated fasting glucose (+32%, *p* < 0.001), total cholesterol (+47%, *p* < 0.001), and triglycerides (+52%, *p* < 0.001) compared with control APP/PS1 littermates. Circulating insulin levels increased nearly fourfold (0.9 ± 0.02 vs. 3.3 ± 0.2 mg/dL; *p* < 0.001), leading to a fivefold elevation in the HOMA-IR index (0.2 ± 0.02 vs. 1.1 ± 0.03; *p* < 0.001). Importantly, no significant differences were observed in serum Aspartate Aminotransferase (AST) or alkaline phosphatase levels between groups, suggesting that fructose-induced metabolic alterations occurred in the absence of detectable changes in conventional markers of liver injury.

Taken together, these results demonstrate that eight weeks of fructose supplementation in APP/PS1 mice is sufficient to induce systemic metabolic stress characterized by hyperglycemia, dyslipidemia, and insulin resistance, closely mimicking a metabolic syndrome-like condition that may contribute to the exacerbation of Alzheimer-related pathology in subsequent analyses.

### 2.2. Fructose Intake Impairs Cognitive Performance and Increases Amyloid Pathology in APP/PS1 and WT Mice

**Figure 2:**
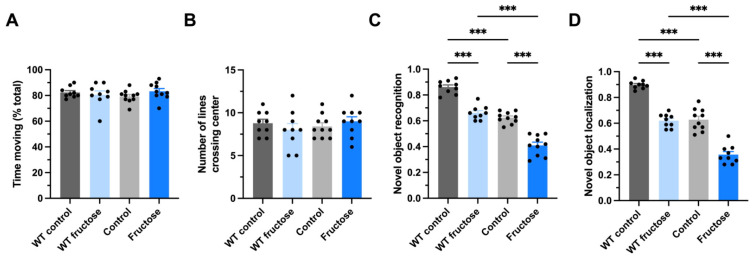
Fructose intake impairs recognition and spatial memory in both WT and APP/PS1 mice without affecting locomotor activity. Behavioral assessment of locomotion, anxiety-like behavior, and cognitive performance in WT and APP/PS1 mice exposed to water or fructose. (**A**) Percentage of time spent moving during the open field test. (**B**) Number of center crossings in the open field arena. (**C**) Discrimination index in the novel object recognition (NOR) test. (**D**) Discrimination index in the novel object location (NOL) test. Fructose-treated APP/PS1 mice showed a significant reduction in recognition and spatial memory compared to water-treated APP/PS1 controls. WT mice exposed to fructose also exhibited a significant decline compared to WT controls, although impairment was more pronounced in the transgenic group. Data are expressed as mean ± SEM. Statistical analysis was performed using Two-way ANOVA (genotype × treatment), *** *p* < 0.001.

Locomotor activity was first evaluated to determine whether fructose exposure affected general motor behavior. In the open field test, no significant differences were observed between water and fructose-treated animals within either genotype in total distance traveled (Figure 2A) or in center exploration (Figure 2B). These results indicate that fructose intake did not modify locomotor activity or anxiety-like behavior in WT or APP/PS1 mice.

Cognitive performance was then assessed using the NOR and NOL tasks. In the NOR test (Figure 2C), fructose-treated APP/PS1 mice showed a significant reduction in the discrimination index compared to water-treated APP/PS1 controls (*p* < 0.001). WT mice exposed to fructose also exhibited a significant decrease in discrimination index compared to WT controls. However, the lowest performance values were observed in fructose-treated APP/PS1 mice.

Similarly, in the NOL task (Figure 2D), fructose-treated APP/PS1 mice displayed a significant reduction in preference for the relocated object compared to water-treated transgenic animals (*p* < 0.001). WT mice receiving fructose also showed a significant decline relative to WT controls. As in the NOR test, the reduction was more pronounced in the APP/PS1 fructose group.

**Figure 3:**
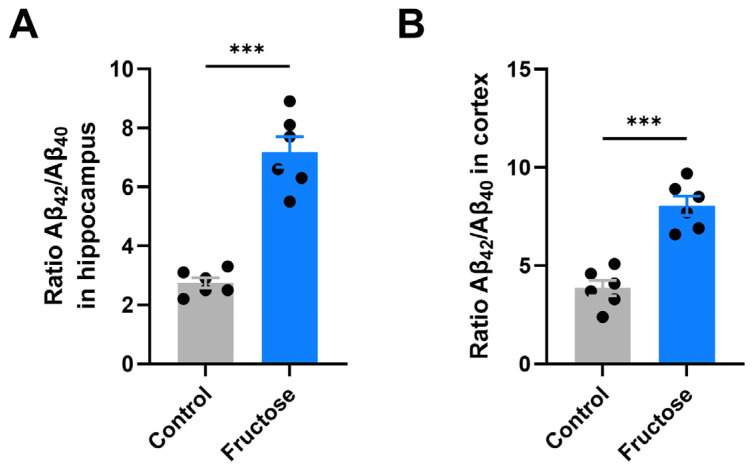
Fructose consumption increases the Aβ_1-42_/Aβ_1-40_ ratio in the hippocampus and cortex. Amyloid-β profile in brain regions of APP/PS1 mice following fructose intake. (**A**) Ratio of Aβ_1-42_/Aβ_1-40_ in the hippocampus. (**B**) Ratio of Aβ_1-42_/Aβ_1-40_ in the cerebral cortex. Fructose-treated mice displayed a marked increase in the neurotoxic Aβ_1-42_/Aβ_1-40_ ratio in both regions. Data are shown as mean ± SEM. Statistical comparisons were performed using an unpaired Student’s *t*-test. *** *p* < 0.001 vs. Control.

Given the association between metabolic stress and amyloidogenesis, we next examined Aβ peptide composition in the hippocampus and cortex (Figure 3). Quantification of the Aβ1-42/Aβ1-40 ratio revealed a robust increase in fructose-fed mice relative to controls in both brain regions (*p* < 0.001; Figure 3A,B). Specifically, the ratio of Aβ1-42/Aβ1-40 nearly tripled in the hippocampus and more than doubled in the cortex, suggesting enhanced amyloidogenic processing following fructose treatment. As a control, we measured the Aβ42/Aβ40 ratio in WT mice and observed significantly lower levels compared to transgenic animals.

Together, these data suggest that fructose consumption is associated with an exacerbation in APP/PS1 mice by exacerbating amyloid accumulation and impairing cognitive functions dependent on hippocampal integrity. This supports the notion that early-life or midlife metabolic disturbances can potentiate neurodegenerative vulnerability, reinforcing the importance of dietary prevention strategies to mitigate AD risk.

### 2.3. Fructose Exposure Reduces Cerebral Glucose Accumulation in Both WT and APP/PS1 Mice, with Greater Impairment in the Transgenic Genotype

**Figure 4:**
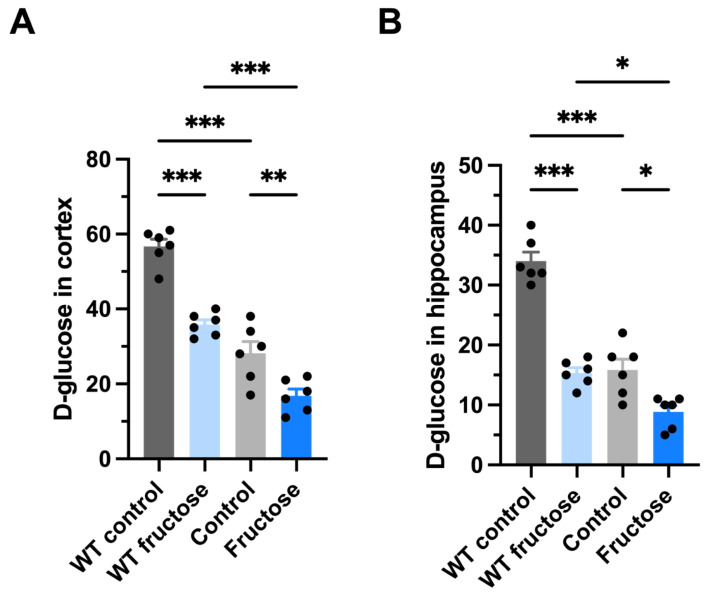
Radiolabeled D-[^14^C]-glucose uptake/accumulation in cortex and hippocampus was measured 30 min after intravenous administration. Fructose intake reduces brain glucose accumulation in the cortex and hippocampus in WT and APP/PS1 mice. Cerebral glucose accumulation measured by radiolabeled D-glucose in WT and APP/PS1 mice following water or fructose exposure. (**A**) D-glucose levels in the cerebral cortex. (**B**) D-glucose levels in the hippocampus. Fructose-treated APP/PS1 mice showed a significant reduction in glucose accumulation compared to water-treated APP/PS1 controls. WT mice exposed to fructose also exhibited reduced glucose levels relative to WT controls in both brain regions. Data are presented as mean ± SEM. Statistical analysis was performed using Two-way ANOVA (genotype × treatment). * *p* < 0.05, ** *p* < 0.01, *** *p* ≤ 0.001 vs. respective control. This assay quantifies tracer uptake/retention and does not reflect absolute brain glucose concentration.

To evaluate whether fructose-induced metabolic alterations affected brain glucose handling across genotypes, we measured glucose accumulation in the cortex and hippocampus using radiolabeled D-glucose. It should be noted that this assay reflects tracer accumulation and does not directly measure glucose transport or absolute tissue glucose levels. In the cortex (Figure 4A), fructose-treated APP/PS1 mice exhibited a significant reduction in glucose accumulation compared with water-treated APP/PS1 controls (*p* < 0.05). As described previously, WT mice exposed to fructose also showed a decrease relative to WT controls [13]. Notably, glucose accumulation was lowest in the fructose-treated APP/PS1 group.

A similar pattern was observed in the hippocampus (Figure 4B). Fructose intake significantly reduced glucose accumulation in APP/PS1 mice compared with transgenic controls (*p* < 0.01). WT mice receiving fructose also displayed reduced accumulation of glucose compared to WT controls, although values remained higher than those observed in fructose-treated APP/PS1 mice.

These results demonstrate that fructose exposure decreases cerebral glucose accumulation in both genotypes, with a more pronounced reduction observed in APP/PS1 animals. The lower tracer accumulation observed in APP/PS1 mice is consistent with the well-established cerebral glucose hypometabolism described in Alzheimer’s disease patients and in multiple AD mouse models [22,23,24,25].

**Figure 5:**
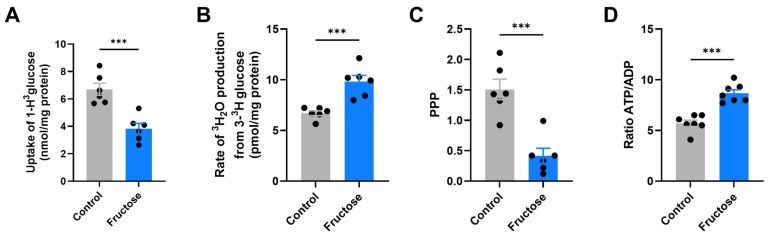
Fructose promotes a metabolic shift toward glycolysis and reduces PPP activity. Effects of fructose on cerebral glucose metabolism in brain hippocampal slices from APP/PS1 mice. (**A**) Uptake of [1-^3^H]-glucose. (**B**) Rate of glycolysis estimated by ^3^H_2_O production from [3-^3^H]-glucose. (**C**) PPP activity. (**D**) Cellular energy status expressed as the ATP/ADP ratio. Fructose intake reduced glucose uptake and PPP activity while enhancing glycolytic flux and ATP/ADP ratio, indicating metabolic reprogramming. Data are shown as mean ± SEM. Statistical significance was assessed using an unpaired Student’s *t*-test. *** *p* < 0.001 vs. Control.

Next, using brain hippocampal slices, we investigated specific pathways of glucose metabolism. Fructose-fed mice showed a significant decrease in total glucose uptake as measured by [1-^3^H]-glucose incorporation (*p* < 0.001; Figure 5A), while the rate of ^3^H_2_O production from [3-^3^H]-glucose, an indicator of glycolytic flux, was markedly increased (*p* < 0.001; Figure 5B). Conversely, the activity of the PPP was significantly reduced (*p* < 0.001; Figure 5C), suggesting a metabolic shift favoring glycolysis over antioxidant and biosynthetic routes. This reprogramming was accompanied by a robust elevation in the ATP/ADP ratio (*p* < 0.001; Figure 5D), reflecting enhanced energy production despite decreased glucose uptake.

### 2.4. Fructose Alters Enzymatic Activities Linked to Energy Homeostasis and the Expression of Inflammation-Related Genes

**Figure 6:**
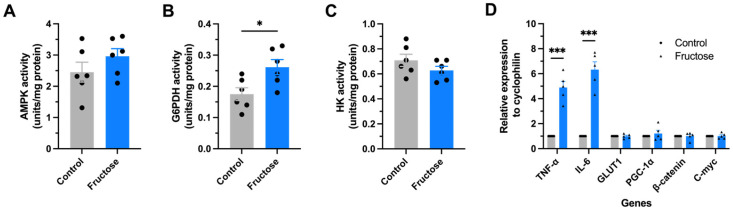
Fructose modulates key metabolic enzyme activities in the brain. Enzymatic activities related to energy metabolism in the brain tissue of hippocampal slices from APP/PS1 mice. (**A**) AMPK activity. (**B**) G6PDH activity. (**C**) HK activity. (**D**) Relative mRNA expression of metabolic and inflammatory markers in brain tissue. Data are expressed as mean ± SEM. Statistical comparisons were performed using an unpaired Student’s *t*-test. * *p* ≤ 0.05, *** *p* < 0.001 vs. Control.

We next assessed the activity of key enzymes regulating energy metabolism in slices from the hippocampus. The activity of AMP-activated protein kinase (AMPK), a master energy sensor, was not significantly different between groups (Figure 6A). However, activity of Glucose-6-phosphate dehydrogenase (G6PDH), the rate-limiting enzyme of the PPP, exhibited a modest but significant increase in fructose-fed mice (*p* < 0.05; Figure 6B), possibly reflecting a compensatory mechanism to counteract oxidative imbalance. In contrast, the activity of Hexokinase (HK) remained unchanged (Figure 6C). These results suggest that although glycolytic entry points and AMPK signaling are preserved, fructose shifts the relative contribution of glucose pathways toward ATP-generating processes rather than redox-regulatory functions.

To explore whether these metabolic alterations were associated with inflammatory responses, we quantified mRNA levels of selected genes in cortical homogenates. Fructose-fed APP/PS1 mice exhibited a striking upregulation of TNF-α (~5-fold) and IL-6 (~6-fold) mRNA levels, indicating activation of proinflammatory transcriptional pathways. Expression of genes associated with glucose transport (GLUT1) and mitochondrial biogenesis (PGC-1α) remained unchanged, as did canonical Wnt-related transcriptional targets (β-catenin and c-myc) (Figure 6D).

## 3. Discussion

Importantly, these findings indicate that fructose exposure, within a genetically vulnerable background, exacerbates early Alzheimer-like features beyond baseline transgene-associated alterations, supporting a synergistic interaction between metabolic stress and neurodegenerative susceptibility. Our results show that fructose exposure is associated with systemic metabolic dysfunction and is accompanied by exacerbation of early Alzheimer-like alterations, including cognitive impairment, increased Aβ_1-42_ accumulation, reduced cerebral glucose utilization, and enhanced neuroinflammatory markers.

Growing evidence supports a strong link between metabolic dysfunction and AD risk. Disturbances in insulin signaling, glucose handling, and lipid metabolism are consistently associated with cognitive decline [26,27]. Although fructose-fed animals exhibited increased body weight, the observed central alterations cannot be solely attributed to weight gain. Previous studies in WT rodent models have shown that fructose intake can induce metabolic, synaptic, inflammatory, and cognitive alterations even in the absence of marked body-weight differences, supporting the idea that fructose-related effects are not exclusively explained by obesity or weight gain [13,15,28].

Moreover, the use of a genetically defined Alzheimer-prone model allows us to specifically evaluate how metabolic stress interacts with pre-existing neurodegenerative vulnerability, rather than acting as an isolated consequence of obesity. Reduced cerebral glucose accumulation is a hallmark of AD and has been documented by FDG-PET both in patients and in transgenic models such as APP/PS1 mice [22,23,29]. Accordingly, the lower tracer accumulation observed in our transgenic animals aligns with previous reports of early metabolic impairment. Given the high energetic demands of the brain, systemic metabolic stress or impaired cerebral glucose transport may accelerate synaptic dysfunction and oxidative stress, thereby contributing to neurodegenerative progression [30,31].

Importantly, even an 8-week exposure to fructose was sufficient to induce insulin resistance and dyslipidemia in APP/PS1 mice, paralleling observations in humans consuming high-sugar diets [32,33]. Although correlation analyses were not formally performed, the parallel changes observed between systemic metabolic parameters (Table 1) and behavioral and neuropathological outcomes (Figure 2 and Figure 3) suggest a potential association between the degree of metabolic dysfunction and the severity of neurological impairment. Future studies should formally address these relationships.

Although fructose-treated mice exhibited increased fluid intake, solid food consumption remained unchanged, indicating that generalized hyperphagia was not present [34]. Moreover, fructose exerts specific metabolic effects independent of caloric load due to its distinct biochemical processing. Unlike glucose, fructose bypasses key regulatory steps in glycolysis and promotes hepatic lipogenesis and insulin resistance even when total caloric intake is controlled [10]. Human intervention studies have shown that fructose-sweetened beverages, but not glucose-sweetened beverages, increase visceral adiposity, triglycerides, and insulin resistance [35]. In addition, sugar-sweetened beverages are strongly associated with cardiometabolic risk independently of overall energy intake [36,37]. Thus, while palatability may have contributed to higher liquid intake, the systemic and cerebral alterations observed here are consistent with fructose-driven metabolic reprogramming within the context of diet-induced metabolic stress, rather than purely hedonic overconsumption.

At the cerebral level, fructose exposure was associated with reduced glucose accumulation and diminished PPP activity, suggesting impaired metabolic flexibility. Despite reduced glucose accumulation, glycolytic flux and ATP/ADP ratio were increased, revealing an “energy paradox”: enhanced ATP generation through glycolysis at the expense of oxidative and antioxidant pathways. The observed increase in glycolytic flux, combined with reduced PPP activity, suggests a shift in glucose utilization that may limit NADPH production. This imbalance could compromise antioxidant defenses and promote oxidative stress, which is a well-established contributor to neurodegeneration and AD pathology. Given that the PPP is a major source of NADPH required for redox homeostasis, its reduction may enhance vulnerability to oxidative damage. In parallel, increased glycolytic flux may reflect a compensatory or maladaptive metabolic response to impaired mitochondrial function. These metabolic alterations may precede or co-occur with both amyloid accumulation and inflammatory activation, supporting the idea that impaired brain energy metabolism may act as a central upstream driver of multiple pathological processes. Mechanistically, fructose bypasses the rate-limiting phosphofructokinase step in glycolysis. After entering cells via GLUT5 and GLUT2 transporters, fructose is phosphorylated by ketohexokinase to fructose-1-phosphate, promoting rapid substrate flux into downstream pathways [38,39,40]. Experimental studies have confirmed fructose transport and metabolism in neurons, astrocytes, and endothelial cells [9,41]. Under high-fructose conditions, astrocytes may convert fructose into lactate, potentially altering astrocyte–neuron metabolic coupling [42,43,44]. Moreover, fructose metabolism has been associated with mitochondrial dysfunction and increased reactive oxygen species (ROS) production [45,46,47] processes highly relevant to AD pathophysiology.

The reduction in PPP activity observed here is particularly significant. The PPP generates NADPH, which is essential for glutathione recycling and membrane repair [48,49]. A decrease in this pathway, combined with increased glycolytic throughput, may exacerbate oxidative stress and lipid peroxidation. Indeed, we previously showed that fructose-induced metabolic syndrome increases lipid peroxidation and disrupts synaptic integrity in WT mice [13]. The present study extends those findings by demonstrating that the same dietary challenge exacerbates amyloid-related and inflammatory alterations in an AD-vulnerable genotype. 

Interestingly, although total PPP flux was reduced, G6PDH activity showed a modest increase, possibly reflecting a compensatory response to oxidative stress. However, this upregulation was insufficient to restore redox balance. AMPK activity remained unchanged, suggesting that canonical energy-sensing pathways are preserved but potentially overridden by substrate-driven metabolic fluxes or inflammatory signaling. The apparent dissociation between reduced glucose uptake and increased glycolysis can be interpreted within the framework of fructose metabolism. By entering glycolysis downstream of phosphofructokinase-1, fructose promotes rapid generation of triose phosphates and enhanced glycolytic throughput [10,50].

In parallel, fructose is more reactive than glucose in promoting non-enzymatic glycation and advanced glycation end-product (AGE) formation. Its rapid conversion into triose phosphates increases the intracellular production of reactive dicarbonyls such as methylglyoxal, a potent inducer of carbonyl and oxidative stress [51,52]. Elevated methylglyoxal has been implicated in insulin resistance and mitochondrial dysfunction, mechanisms relevant to both metabolic syndrome and AD. Together with the observed increase in ATP/ADP ratio [53,54]. These findings support a fructose-driven redistribution of carbon flux toward ATP-generating pathways at the expense of NADPH-producing routes, potentially increasing vulnerability to chronic oxidative or inflammatory stress.

Metabolic dysregulation and inflammation are tightly interconnected. Insulin resistance and elevated lipids can activate toll-like receptor signaling and NF-κB pathways, promoting the release of TNF-α and IL-6 [55,56,57]. These cytokines impair insulin signaling and glucose transport in the brain, creating a self-reinforcing cycle of metabolic and inflammatory dysfunction [58]. In our study, fructose-fed APP/PS1 mice exhibited a marked increase in TNF-α and IL-6 mRNA levels, whereas genes related to mitochondrial biogenesis and Wnt signaling remained unchanged. Elevated TNF-α can enhance amyloidogenic processing of APP, and IL-6 may promote Aβ aggregation and impair clearance, mechanisms consistent with the increased Aβ_1-42_/Aβ_1-40_ ratio observed in cortex and hippocampus. These metabolic alterations may contribute to amyloidogenic processing and neuroinflammatory responses, linking altered energy metabolism with key pathological hallmarks of AD. However, the present study does not allow us to establish the temporal sequence or causal order between neuroinflammation and amyloid pathology. It remains unclear whether fructose-induced inflammatory activation promotes amyloidogenic processing of APP, or whether early alterations in amyloid metabolism and cerebral energy balance trigger secondary inflammatory responses. Both scenarios are supported by previous literature and are not mutually exclusive [59,60,61,62]. An alternative and potentially unifying interpretation is that metabolic dysfunction represents an upstream event driving both processes in parallel. In this framework, fructose-induced alterations in glucose metabolism, insulin signaling, and redox balance may simultaneously promote amyloidogenic pathways and inflammatory responses, leading to a feed-forward cycle that exacerbates neurodegenerative vulnerability [63,64]. It should be noted that inflammatory markers were assessed at the mRNA level, which reflects transcriptional activation but does not necessarily correspond to protein abundance or cytokine secretion. However, increased TNF-α and IL-6 transcription is widely recognized as an early indicator of neuroinflammatory activation and has been consistently associated with functional inflammatory responses in similar experimental models [65,66,67].

The preferential increase in Aβ_1-42_ is particularly relevant, as this isoform is more aggregation-prone and neurotoxic than Aβ1-40 [68]. An elevated Aβ_1-42_/Aβ_1-40_ ratio accelerates plaque formation and synaptic dysfunction [69]. Previous studies have shown that high-fat or high-sugar diets exacerbate amyloid pathology through metabolic and inflammatory pathways [70,71]. Our data demonstrate that fructose exposure, in the absence of detectable alterations in standard serum markers of liver injury, is associated with increased Aβ_1-42_/Aβ_1-40_ ratio and metabolic dysfunction. These findings suggest that fructose accelerates the convergence of metabolic, oxidative, and inflammatory insults that compromise synaptic resilience. This may be particularly detrimental during early disease stages, when compensatory mechanisms are still active. Therefore, interventions targeting metabolic health during pre-symptomatic phases may offer significant preventive potential. From a translational perspective, strategies aimed at improving insulin sensitivity or reducing inflammation may complement dietary interventions. However, prevention of metabolic dysfunction remains the most effective approach, underscoring the importance of limiting added sugars in modern diets.

Several limitations should be acknowledged. A key limitation of the present study is the absence of an isocaloric glucose control or pair-fed design, which prevents us from definitively attributing the observed effects to fructose-specific mechanisms. Therefore, we cannot exclude the possibility that the metabolic disturbances induced by increased caloric intake or systemic insulin resistance, rather than fructose per se, contribute to the observed neurological alterations. 

In addition, inflammatory markers were evaluated at the mRNA level only, and future studies incorporating protein-level measurements or cytokine secretion assays would provide a more comprehensive characterization of the neuroinflammatory response.

In addition, the cross-sectional design of the study does not allow determination of the temporal sequence between metabolic dysfunction, amyloid pathology, and neuroinflammation.

The exposure period was relatively short, and longer treatments may reveal progressive or irreversible effects. Only male mice were analyzed, and sex-specific responses warrant further investigation. In addition, we focused primarily on amyloid-related mechanisms; future studies should examine tau pathology, synaptic proteomics, and neurovascular alterations. The absence of an isocaloric glucose control or pair-fed design also limits the ability to fully dissociate fructose-specific effects from caloric contribution. Although ad libitum fructose administration mimics real-world exposure to sugar-sweetened beverages, future studies incorporating caloric matching strategies will be important to further dissect substrate-specific mechanisms. Importantly, this limitation also highlights that diet-induced metabolic dysfunction itself may represent a central driver of Alzheimer-like alterations, independently of the specific nutrient source. Nevertheless, our aim was to model a clinically relevant scenario of chronic fructose overconsumption, and within this framework, the key finding remains that metabolic stress exacerbates Alzheimer-like pathological and inflammatory outcomes in an AD-vulnerable model [72].

Taken together, our findings support a model in which diet-induced metabolic stress, exemplified here by fructose consumption, acts as a key driver that amplifies early Alzheimer-related alterations in a susceptible brain, linking peripheral metabolic dysfunction with central neurodegenerative processes. These results reinforce the concept that lifestyle factors, particularly diet, may critically modulate disease onset and progression, highlighting metabolic regulation as a potential target for early intervention in AD.

## 4. Materials and Methods

### 4.1. Animals and Treatments

For an AD model, we used male APPswe/PS1dE9 (RRID: MMRRC_34829-JAX, 6-month-old, APP/PS1) as an asymptomatic model before starting the treatment. APP/PS1 animals co-express the Swedish (K594M/N595L) mutation of a chimeric mouse/human APP (Mo/HuAPP695swe) together with the human exon-9-deleted variant of PS1 (PS1-dE9); these mice secrete elevated levels of human Aβ peptide [19]. This strain was obtained from The Jackson Laboratory (USA). We established two groups of animals, each comprising 12 mice; both groups were fed a control diet (Control, 10 % of fat in diet, TestDiet, USA). One group received water, whereas the second group received water supplemented with 15% fructose. To isolate the effect of fructose exposure within a genetically defined AD-vulnerable background, analyses were designed as within-genotype comparisons (APP/PS1 fructose vs. APP/PS1 water). Direct comparisons between transgenic animals and independently sourced WT controls can be confounded by genetic background/substrain differences and baseline genotype-dependent phenotypes, potentially leading to misleading conclusions. Accordingly, rigorous phenotyping of genetically modified mice is recommended to rely on appropriately matched controls (ideally littermate/background-matched) when quantifying treatment effects [73,74]. In addition, WT C57BL/6 male mice were included as reference cohorts for selected endpoints (behavioral testing, brain glucose accumulation, and Aβ_42_/Aβ_40_ ratio). WT mice (6 months old) were assigned to two groups (*n* = 6/group): water (WT-Control) or 15% fructose in drinking water (WT-Fructose) for 8 weeks, under the same exposure conditions. WT data were used to determine whether fructose effects observed in APP/PS1 mice were also present in non-transgenic animals; direct genotype comparisons were not used as primary treatment-effect estimates due to potential baseline genotype and background-related differences. Where appropriate, controls were interpreted within each genotype. The Committee for Ethics of Animal Experiments approved the experiments, which were carried out in accordance with the Guidelines for Animal Experiments, P. Universidad Católica de Chile, and the Manual of Biosafety Standards and Associated Risks, CONICYT 2009.

### 4.2. Biochemical Analysis

MetS involves a conglomerate of pathological features, including obesity, insulin resistance, hypertension, high triglyceride levels, cardiovascular disease, and systemic inflammation [75]. Accordingly, we measured several of these features in the animals exposed to fructose. Blood was collected from the tail vein after 6 h of fasting, and then the serum samples were obtained. Glucose levels were measured according to the hexokinase/G-6-PDH method, using Architect Analyzer (Abbott Laboratories, Abbott Park, IL, USA), and insulin levels were measured via chemiluminescence (Beckman Coulter); in both cases, the manufacturers’ instructions were followed. The HOMA, an index of insulin resistance, was calculated using the following formula: HOMA-R = fasting glucose (mmol l^−1^) × fasting insulin (μIU ml^−1^)/22.5. The levels of triglycerides and cholesterol were assessed enzymatically using the Architect c8000 analyzer (Abbott Laboratories, Abbott Park, IL). Alkaline phosphatase activity was measured in the stool supernatant using an automatic biochemistry analyzer. For this, 20 μl of supernatant was added to 1 ml of assay buffer containing p-nitro-phenyl phosphate as a substrate and incubated for 1 min at 37° C. The resulting Alkaline phosphatase activity was then quantified by the analyzer, which was previously calibrated with Alkaline phosphatase standards. AST was measured using an automatic blood chemical analyzer (Hitachi, Tokyo, Japan) [76]. 

### 4.3. Large Open-Field (LOF) Test

A 120 × 120 cm transparent Plexiglas cage with 35-cm-high transparent walls was used to study locomotor and stress behavior in our groups. The open field, which measured 40 × 40 cm, was defined as the “center” area of the field. Data were collected using an automatic tracking system (HVS Imagen, Bicester, UK). Each mouse was placed alone in the center of the open field, and its behavior was tracked for 20 min. At the end of the session, the mouse was returned to its home cage. The parameters measured included the total time moving and the number of times the mouse crossed the center area of the platform [77]. 

### 4.4. Novel Object Recognition and Novel Object Location

The novel object recognition (NOR) and novel object location (NOL) tasks were performed as previously described [78,79]. Mice were habituated to the experimental room in the experimental cages for three consecutive days for 30 min per day (three consecutive days) and for 1 h on the testing day. The task was conducted on a 120 × 120 cm transparent Plexiglas platform with 35-cm-high transparent walls containing two identical objects placed at specific locations. For object familiarization, mice were allowed to explore the platform for 10 min. The animals were subsequently returned to their home cages for 1 h, followed by a 5 min exposure to a novel localization of one of the familiar objects (NOL) or a new object (NOR). The mice were again returned to their home cages for 1 h and were subsequently exposed to a novel object for 5 min. The mice had no observed baseline preference among the different objects. 

Memory performance in the NOR and NOL tasks was quantified using a Preference Index, calculated as the time spent exploring the novel object or the object in the new location divided by the total exploration time of both familiar and novel objects. This index is commonly used to normalize exploration behavior and to control for inter-animal variability in total exploration time. Cognitively intact mice typically display a robust preference for novelty, whereas impairments in recognition or spatial memory are reflected by a reduced preference index [80,81]. The cages were routinely cleaned with ethanol following mouse habituation and testing [78].

### 4.5. Detection and Quantification of Aβ Peptide Levels

Two sandwich ELISAs specific for Aβ_1-40_ and Aβ_1-42_ were employed to determine the concentrations of Aβ peptides, as previously described [29]. Hippocampal and cortical homogenates from each animal were diluted to 1 μg/μl in homogenization buffer containing protease and phosphatase inhibitors. According to the manufacturer’s instructions, diluted homogenates (100 μl) were prepared to measure Aβ_1-42_/Aβ_1-40_ levels. Plates were read at the corresponding wavelengths on a Metertech (Taipei City, Taiwan) 960 ELISA Analyzer.

### 4.6. D-[1-^14^C] Glucose Biodistribution

Upon completing cognitive tests, some mice from each group were injected with D-[1-^14^C] glucose (#NEC043, PerkinElmer, Waltham, MA, USA) via the tail vein. Briefly, mice were anesthetized with isoflurane and injected intravenously via the tail with 50 mCi of tracer diluted to a final volume of 20 mL in isotonic saline (from this solution, we injected 25–40 mL into each animal, depending on the weight of each animal). Following a 15 min accumulation period, the animals were euthanized and tissues were collected. Tissue radioactivity was quantified by liquid scintillation. D-[1-^14^C] glucose levels were normalized to the weight of resected tissue and expressed as the percent of the injected dose [82,83].

### 4.7. Hippocampal Slices Preparation 

Hippocampal slices were prepared as previously described [84]. Briefly, transverse slices (350 μm) from the dorsal hippocampus were sectioned in cold artificial cerebrospinal fluid (119 mM NaCl, 26.2 mM NaHCO_3_, 2.5 mM KCl, 1 mM NaH_2_PO_4_, 1.3 mM MgCl_2_, 10 mM glucose, 2.5 mM CaCl_2_) and incubated in artificial cerebrospinal fluid for 1 h at 22°C before using.

### 4.8. Glucose Uptake Analysis

After the respective treatment slices were washed twice with incubation buffer (135 mM NaCl, 15 mM HEPES, 5 mM KCL, 1,8 mM CaCl_2_ and 0,8 mM MgCl_2_) and 1-1.2 μCi of Deoxy-D-glucose, 2-[1,2-^3^H(N)] ([^3^H]-2-DG) was added for 1-3 times. Glucose uptake was then arrested with detention buffer (add 0,2 mM HgCl_2_ to incubation buffer (pH 7.4) and cells were lysed in 1 ml of lysis buffer (10 mM Tris-HCL and 0,2% SDS, pH 8.0). Then, 3 ml of scintillating solution were added to the cell lysates and radioactivity was measured using a liquid scintillation counter (TriCarb 2900TR analyzer). Mercuric chloride (HgCl_2_) was included in the stop solution to rapidly and irreversibly inhibit glucose transport by binding to thiol groups in membrane transport proteins, thereby preventing any further uptake of glucose or glucose analogues during sample processing. Mercurial compounds are well-known inhibitors of GLUT-mediated transport and have been used in glucose uptake assays to ensure an immediate termination of transporter activity [85,86].

### 4.9. Determination of the Glycolytic Rate

Glycolytic rates were determined as previously described [29]. Briefly, slices were placed in tubes containing 5 mM glucose and then washed twice in Krebs–Henseleit solution (11 mM Na_2_HPO_4_, 122 mM NaCl, 3.1 mM KCl, 0.4 mM KH2PO4, 1.2 mM MgSO_4_, and 1.3 mM CaCl_2_, pH 7.4) containing the appropriate concentration of glucose. After equilibration in 0.5 mL of Hank’s balanced salt solution/glucose (cat: 14025076, ThermoFisher-Waltham, MA, USA) at 37 °C for 30 min, 0.5 mL of Hank’s balanced salt solution containing various concentrations of [3-^3^H] glucose (cat: NET331C250UC, PerkinElmer, USA) was added, with a final specific activity of 1–3 disintegrations/min/pmol (~1 mCi/mmol). Aliquots of 100 μL were then transferred to another tube, placed inside a capped scintillation vial containing 0.5 mL of water, and incubated at 45 °C for 48 h. After this vapor-phase equilibration step, the tube was removed from the vial, the scintillation mixture was added, and the 3H2O content was determined by counting over a 5-min period.

### 4.10. Measurement of Glucose Oxidation Through the PPP

Glucose oxidation through the PPP was quantified following established radiometric methods, based on the differential decarboxylation of [1-^14^C]-glucose and [6-^14^C]-glucose. Briefly, [1-^14^C]-glucose releases ^14^CO_2_ both in the 6-phosphogluconate dehydrogenase reaction of the PPP and in the Krebs cycle, whereas [6-^14^C]-glucose is decarboxylated exclusively in the Krebs cycle. Thus, PPP-derived glucose oxidation was calculated as the difference in ^14^CO_2_ production between both labeled substrates. Slices were washed with ice-cold PBS, harvested, and immediately transferred into O_2_-saturated Krebs–Henseleit buffer (122 mM NaCl, 3.1 mM KCl, 0.4 mM KH_2_PO_4_, 11 mM Na_2_HPO_4_, 1.2 mM MgSO_4_, 1.3 mM CaCl_2_, pH 7.4). The cellular suspension was placed in Erlenmeyer flasks containing an additional 0.5 mL of Krebs–Henseleit buffer supplemented with either 0.5 μCi D-[1-^14^C]-glucose or 2 μCi D-[6-^14^C]-glucose, together with unlabeled D-glucose to reach a final concentration of 5.5 mM. Each flask was fitted with a central well containing an Eppendorf tube with 500 μL benzethonium hydroxide to trap the released ^14^CO_2_. Flasks were flushed with O_2_ for 20 s, sealed with rubber caps, and incubated in a shaking water bath at 37 °C for 60 min. Reactions were terminated by injecting 0.2 mL of 1.75 M HClO_4_ into the main chamber to release dissolved CO_2_. Shaking was continued for an additional 20 min to ensure complete trapping of ^14^CO_2_. The radioactivity accumulated in the benzethonium hydroxide was quantified by liquid scintillation counting. PPP-dependent glucose oxidation was obtained by subtracting the ^14^CO_2_ values generated from [6-^14^C]-glucose from those generated from [1-^14^C]-glucose. Radiolabeled substrates were purchased from PerkinElmer (Massachusetts, USA) 32,43.

### 4.11. Quantification of ADP and ATP Levels

ATP and ADP levels were measured using an ATP determination kit [#A22066, Invitrogen/Molecular Probes] or an ADP assay Kit [#ab83359, Abcam, Cambridge, UK], respectively [87,88]. The ADP/ATP ratio was measured accordingly.

### 4.12. Hexokinase Activity

To measure the hexokinase activity, the hippocampal slices were washed with PBS, treated with trypsin/EDTA, and centrifuged at 500g for 5 min at 4 ◦ C. Lysates were then resuspended in isolation medium (250 mM sucrose, 20 mM HEPES, 10 mM KCl, 1.5 mM MgCl2, 1 mM EDTA, 1 mM DTT, 2 mg/ml aprotinin [#A1153], 1 mg/ml pepstatin A [#77170], and 2 mg/ml leu-peptin [#L8511], reagents from Sigma-Aldrich (St Louis, MO, USA), at a 1:3 dilution, sonicated at 4° C, and then centrifuged at 1500g for 5 min at 4 ◦ C. The supernatant fraction was mixed with the reaction medium (25 mM Tris-HCl, 1 mM DTT, 0.5 mM NADP/Na^+^, 2 mM MgCl_2_, 1 mM ATP, 2 U/ml G6PDH, and 10 mM glucose) and incubated at 37° C for 30 min. The reaction was stopped by the addition of 10% trichloroacetic acid, TCA, [#T6399, Sigma-Aldrich], and the generation of NADPH was measured at 340 nm [29].

### 4.13. Determination of G6PDH Activity

Hippocampal slices were washed with PBS, collected by trypsinization (0.25% trypsin- 0.2% EDTA (*w*/*v*)), and pelleted. The tissue samples were then resuspended in isolation medium (250 mM sucrose, 20 mM HEPES, 10 mM KCl, 1.5 mM MgCl_2_, 1 mM EDTA, 1 mM DTT, 2 mg/ml aprotinin, 1 mg/ml pepstatin A, and 2 mg/ml leupeptin) at a 1:3 dilution, sonicated at 4° C, and centrifuged for 5 min at 1500g at 4 °C. Subsequently, the supernatant was further separated by centrifugation at 13,000g for 30 min at 4◦ C. Finally, the G6PDH activity of the supernatant was quantified in a reaction buffer containing 1 mM ATP and 10 mM glucose-6-phosphate (G6P) for 30 min at 37 ◦ C. The reaction was stopped by the addition of 10% TCA. The generation of NADPH was measured at 340 nm, as described previously [29].

### 4.14. AMPKα Activity

Active (phospho-T172) AMPK was then measured using the AMPK alpha phospho human ELISA kit (KHO0651, Thermo Fisher, Waltham, MA, USA), according to the manufacturer’s instructions and as described previously.

### 4.15. Quantitative Real-Time PCR (qRT-PCR)

RNA was isolated from the hippocampus and reverse transcribed into cDNA [#18091050, Invitrogen]. Quantitative real-time RT–PCR (qRT–PCR) was conducted using SYBR master mix [#4368577, ThermoFisher Scientific], with the program recommended by the manufacturer and as published previously. As a reference, we used the housekeeping gene cyclophilin, and the relative Ct values of each gene were calculated using the delta Ct, in comparison with the control gene. Duplicated control reactions for every sample without reverse transcription were included to ensure that PCR products were not due to the amplification of contaminated genomic DNA. The sets of primers (IDT Integrated DNA Technologies, Coralville, IA, USA) used are the following: GLUT1 (foward: 5′-ATGGATCCCAGCAGCAAGAAG-3′, reverse: 5′-AGAGACCAAAGCGTGGTGAG-3′); c-Myc (foward: 5′-GGAGTGGTTCAGGATTGGGG-3′, reverse: 5′-GGGTAGCTTACCAGAGTCGC-3′), IL-6 (foward: 5′-CAACGATGATGCACTTGCAGA-3′, reverse: 5′-GTGACTCCAGCTTATCTCTTGGT-3′, b-catenin (foward: 5′-TCCTTCACGCAAGAGCAAGTA-3′, reverse: 5′-CGCACCATGCAGAATACAAATGA-3′), TNF-a (foward: 5′-TGATCGGTCCCCAAAGGGAT-3′, reverse: 5′- TGTCTTTGAGATCCATGCCGT-3′), PGC-1a (foward:5′-AGCCGTGACCACTGACAACGAG-3′, reverse: 5′-GCTGCATGGTTCTGAGTGCTAAG-3′) and housekeeping cyclophilin (forward: 5′-TGGAGATGAATCTGTAGGAGGAG-3′, reverse: 5′- TACCACATCCATGCCCTCTAGAA-3). Cyclophilin A was selected as the reference gene because of its well-documented stable expression in brain tissue under both physiological and pathological conditions, including neurodegenerative and metabolic stress models [89,90]. 

### 4.16. Statistical Analysis

All experiments were performed using samples from at least 6 animals per group. Data are presented as mean ± standard error of the mean (SEM). Depending on the experimental design, statistical comparisons were performed using one-way or two-way analysis of variance (ANOVA) followed by Bonferroni’s post hoc test to correct for multiple comparisons. Before running ANOVAs, we verified normality (Shapiro–Wilk test) and homogeneity of variance (Levene’s test) using Prism (https://www.graphpad.com/) (GraphPad, San Diego, CA, USA). In cases where assumptions were not met, data were log-transformed, and the analysis was repeated to ensure robustness. The significance thresholds were set at *p* ≤ 0.05 (*), *p* ≤ 0.01 (**), and *p* ≤ 0.001 (***). Potential outliers were evaluated using the ROUT method (Q = 1%) implemented in Prism; no outliers were detected or removed from the analyses. All statistical settings (alpha = 0.05, confidence interval = 95%) followed Prism’s standard parameters. The complete statistical framework has been clarified to enhance reproducibility and transparency.

## Figures and Tables

**Table 1:** Metabolic and biochemical parameters in APP/PS1 mice after chronic fructose intake.

Treatment/Condition	Control	Fructose
Glucose (mg/dl)	95.2 ± 2.7	125.5 ± 6.1 ***
Cholesterol (mg/dl)	121.7 ± 3.1	179.1 ± 9.3 ***
Triglycerides (mg/dl)	85.6 ± 2.4	129.7 ± 8.5 ***
Insulin (mg/dl)	0.9 ± 0.02	3.3 ± 0.2 ***
HOMA	0.2 ± 0.02	1.1 ± 0.03 ***
Alkaline phosphatase (UI/L)	76.2 ± 2.9	82.7 ± 3.5
AST (UI/L)	86.2 ± 4.3	88.1 ± 2.9

*** *p* < 0.001.

## Data Availability

Data will be made available on request.

## Abbreviations

The following abbreviations are used in this manuscript:

AD	Alzheimer disease
AMPK	AMP-activated protein kinase
Aβ	Beta-amyloid peptide
2-DG	2-deoxyglucose
GLUT1	Glucose transporter 1
G6PDH	Glucose-6-phosphate dehydrogenase
Hk	Hexokinase
NADPH	Adenine dinucleotide phosphate
PPP	Pentose phosphate pathway
ROS	Reactive oxygen species
